# Reply to Lunghi, C.; Baroni, F. Comment on “Tekin et al. Novel Conservative Therapies in Migraine Management: The Impact of Fascia Exercises in a Randomized Controlled Trial. *J. Clin. Med.* 2025, *14*, 539”

**DOI:** 10.3390/jcm15124658

**Published:** 2026-06-16

**Authors:** Rabia Tuğba Tekin, Hilal Aslan, Veysel Uludağ, Şadiye Gümüşyayla, Gönül Vural

**Affiliations:** 1 Faculty of Physiotherapy and Rehabilitation, Hacettepe University, Ankara 06800, Türkiye; 2 Faculty of Health Science, Physiotherapy and Rehabilitation, Ankara Yıldırım Beyazıt University, Ankara 06800, Türkiye; hilalaslan@aybu.edu.tr; 3 Department of Physiotherapy and Rehabilitation, Faculty of Health Sciences, Duzce University, Duzce 81620, Türkiye; vuludag1365@outlook.com; 4 Department of Neurology, Faculty of Medicine, Ankara Yıldırım Beyazıt University, Ankara 06800, Türkiye; sadiyetemel@yahoo.com (Ş.G.); gonulvrl@gmail.com (G.V.)

**Keywords:** musculoskeletal physiological phenomena, movement, motion, manipulation, osteopathic, exercise movement techniques, exercise therapy, mind–body therapies, bodyworks

We would like to sincerely thank Lunghi and Baroni for their thoughtful, constructive, and collegial Comment on our published article entitled “Novel Conservative Therapies in Migraine Management: The Impact of Fascia Exercises in a Randomized Controlled Trial” [1]. We greatly appreciate their careful reading of our work and their valuable effort to clarify the osteopathic origins, conceptual background, and methodological framework of Fascial Pattern Exercises.

We fully acknowledge that the Fascial Pattern Exercises included in our intervention have important conceptual and methodological roots in the osteopathic literature. In particular, we recognize that this approach was previously described by Lunghi, Baroni, and colleagues within the broader context of osteopathic clinical reasoning, patient active-participative osteopathic approaches, and functional neuromyofascial activity [2,3,4,5]. We also acknowledge that the original conceptualization of this approach includes not only the execution of specific movements and postures, but also osteopathic assessment, clinical reasoning, palpatory evaluation of somatic dysfunction, interpretation of fascial patterns, motor skill assessment, and patient-assisted activities within an individualized and person-centered framework [2,3,4,5]. We regret that our original publication did not sufficiently contextualize these osteopathic foundations and did not include all relevant references that would have more clearly acknowledged the development and theoretical background of Fascial Pattern Exercises. This omission was entirely unintentional. We are grateful to Lunghi and Baroni for bringing this issue to our attention in a constructive manner, as their Comment provides an important opportunity to clarify the methodological background of the intervention and to improve the transparency of the scientific discussion.

In our randomized controlled trial, the fascia-specific component was applied as a standardized, physiotherapy-based active exercise protocol in patients with migraine. As described in the Methods section of our article, the intervention included exercises targeting appendicular, axial, meningeal, and visceral fascia, performed in different positions, with repeated movements and sustained end-range postures; archetypal postures were also practiced at the beginning and end of the sessions [6]. However, we would like to clarify that the intervention used in our trial should not be interpreted as a complete osteopathic manipulative treatment protocol. Rather, it represented a structured and standardized exercise-based adaptation of fascia-oriented principles within a physiotherapy research setting.

This distinction is important. As Lunghi and Baroni correctly emphasize, the full osteopathic application of Fascial Pattern Exercises is embedded within a broader clinical framework that includes assessment of somatic dysfunction, identification of specific body regions and generalized fascial patterns, evaluation of motor skills, and the integration of patient-assisted movements and postures with osteopathic manipulative treatment [2,3,4,5]. These components require specific osteopathic competencies and individualized clinical reasoning. In contrast, our study was designed to evaluate the effects of a reproducible, standardized fascia-oriented exercise program in a randomized controlled design. Therefore, individualized osteopathic assessment, palpatory diagnosis of somatic dysfunction, and osteopathic manipulative treatment were not part of the intervention protocol and were not evaluated as study variables. Accordingly, the findings of our study should be interpreted within this specific methodological scope. Our results suggest that a standardized fascia-oriented active exercise program may have beneficial effects on pain intensity, migraine-related disability, sleep quality, psychological symptoms, and patient satisfaction in individuals with migraine [6]. However, these findings should not be considered as evidence for the effectiveness of the complete osteopathic model of Fascial Pattern Exercises or patient active-participative osteopathic approaches. Rather, they provide preliminary evidence regarding a physiotherapy-based implementation of selected fascia-oriented exercise components. We agree that future studies should more explicitly define the relationship between standardized exercise protocols and the broader osteopathic framework from which these methods originate. We also agree with Lunghi and Baroni that accurate attribution is essential for scientific transparency, reproducibility, and appropriate clinical interpretation. Precise referencing is particularly important when interventions are derived from complex multimodal or integrative therapeutic traditions. The works of Lunghi and colleagues provide an important conceptual and practical framework for understanding the distinctive and interprofessional dimensions of these approaches [2,3,4,5,7]. In this regard, their publications on patient active approaches in osteopathic practice, functional neuromyofascial activity, and patient–practitioner–environment synchronization offer valuable context for researchers and clinicians interested in integrating fascia-oriented and person-centered approaches into interdisciplinary care [3,4,5]. We further appreciate the authors’ emphasis on the interprofessional nature of this field. Migraine is a multidimensional neurological disorder involving pain modulation, autonomic regulation, musculoskeletal factors, psychological burden, and behavioral components. For this reason, conservative management strategies may benefit from collaboration among neurologists, physiotherapists, osteopaths, and other healthcare professionals. Future research should consider interprofessional designs in which osteopathic assessment, physiotherapy-based exercise prescription, patient education, and neurophysiological outcome measures are integrated in a clearly defined and methodologically rigorous manner. In addition, future trials should aim to compare different levels of intervention complexity. For example, studies may compare standardized fascia-oriented exercise programs, individualized osteopathic approaches based on somatic dysfunction and fascial pattern assessment, conventional physiotherapy, and combined interprofessional models. Such designs may help clarify which components are most clinically relevant, which patient subgroups may benefit most, and whether individualized osteopathic assessment provides additional benefit beyond standardized exercise-based protocols. Once again, we sincerely thank Lunghi and Baroni for their constructive Comment and for highlighting the need to more clearly acknowledge the osteopathic origins and conceptual background of Fascial Pattern Exercises. We believe that their contribution strengthens the interpretation of our study and supports a more accurate understanding of the intervention used. We hope that this Reply clarifies the scope of our trial, properly recognizes the relevant prior work, and contributes to a respectful and scientifically rigorous dialogue on the role of fascia-oriented, osteopathic, and physiotherapy-based approaches in migraine management.
